# Therapeutic Potential of GABAergic Signaling in Myelin Plasticity and Repair

**DOI:** 10.3389/fcell.2021.662191

**Published:** 2021-04-06

**Authors:** Daniel Reyes-Haro, Abraham Cisneros-Mejorado, Rogelio O. Arellano

**Affiliations:** Instituto de Neurobiología, Universidad Nacional Autónoma de México Campus Juriquilla, Juriquilla, Mexico

**Keywords:** GABA_*A*_ receptors, oligodendrocyte precursor cells, NG2 glia, myelination, remyelination, β-carbolines, neurosteroids

## Abstract

Oligodendrocytes (OLs) produce myelin to insulate axons. This accelerates action potential propagation, allowing nerve impulse information to synchronize within complex neuronal ensembles and promoting brain connectivity. Brain plasticity includes myelination, a process that starts early after birth and continues throughout life. Myelin repair, followed by injury or disease, requires new OLs differentiated from a population derived from oligodendrocyte precursor cells (OPCs) that continue to proliferate, migrate and differentiate to preserve and remodel myelin in the adult central nervous system. OPCs represent the largest proliferative neural cell population outside the adult neurogenic niches in the brain. OPCs receive synaptic inputs from glutamatergic and GABAergic neurons throughout neurodevelopment, a unique feature among glial cells. Neuron-glia communication through GABA signaling in OPCs has been shown to play a role in myelin plasticity and repair. In this review we will focus on the molecular and functional properties of GABA_*A*_ receptors (GABA_*A*_Rs) expressed by OPCs and their potential role in remyelination.

## Introduction

**FIGURE 1:**
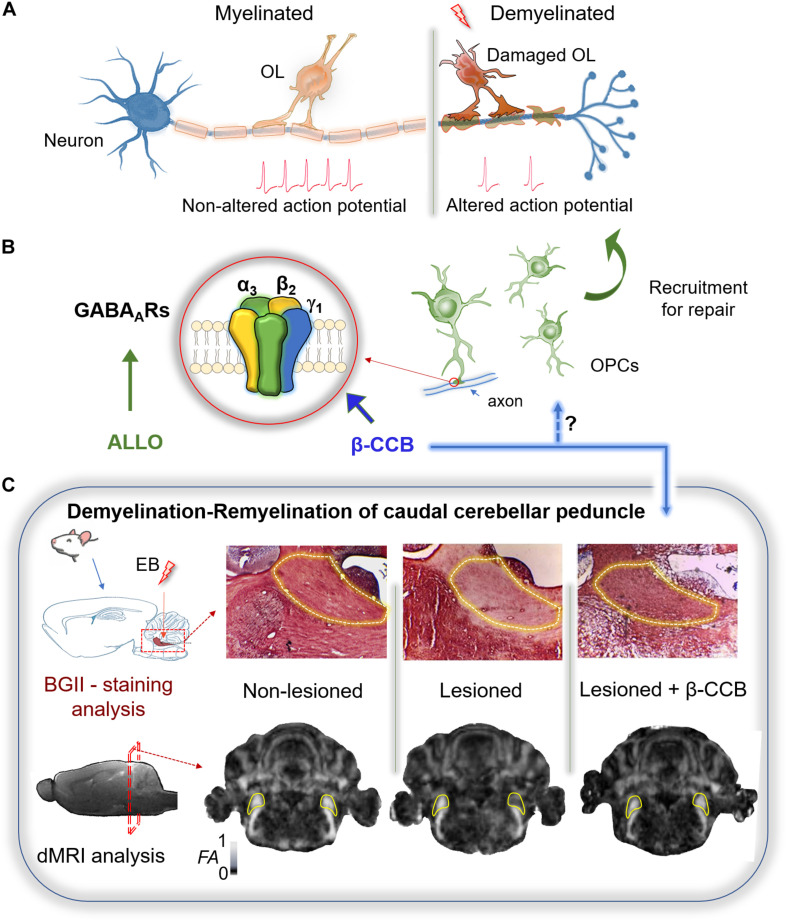
Therapeutic potential of GABAergic signaling on remyelination. **(A)** Myelin sheath accelerates the action potential propagation. The loss of oligodendrocytes (OLs) after white matter injury results in demyelination and altered conduction of the nerve impulse. **(B)** Remyelination requires of OPCs recruitment. These cells express mainly a GABA_*A*_R with a defined subunit arrangement (α3, β2, γ1) that is potentiated by some β-carbolines (particularly β-CCB). Allopregnanolone (ALLO) also modulates positively several GABA_*A*_Rs and has been shown to promote myelination in different models of demyelination. **(C)** Demyelination of the rat caudal cerebellar peduncle (c.c.p.; yellow line area in both tissue and MRI sections) was used as an experimental model to test the effect of β-carbolines on remyelination. Stereological injection of ethidium bromide (EB) in the c.c.p. induced its demyelination. Both black gold II (BGII) staining, and diffusion-weighted MRI (dMRI) analysis, were performed in three groups of animals: non-lesioned (vehicle-injected), lesioned (injected unilaterally in the right c.c.p.), and lesioned that were systemically treated with β-CCB. In the lesioned group both BGII-staining and fractional anisotropy (FA) decayed in the ipsilateral side compared against the control contralateral side. The β-carboline administration promoted remyelination as confirmed by both experimental analyses, suggesting that OPC-GABA_*A*_R positive modulation by β-CCB favors the repairing process (modified from Cisneros-Mejorado et al., 2020).

The oligodendrocyte precursor cells (OPCs) are a dynamic glial population widely distributed in the central nervous system which differentiate into new oligodendrocytes (OLs) participating in myelin remodeling (Serwanski et al., 2018; Bonetto et al., 2020). OPCs express the NG2 antigen and the α receptor for platelet-derived growth factor (PDGFRα) and arise sequentially in three waves during early neurodevelopment (Nishiyama et al., 1999, 2009, 2016; Guo et al., 2021). The first wave derives from Nkx2.1^+^ progenitors (E12.5) from the ganglionic eminence and anterior entopeduncular area in the ventral brain. The second wave (E16.5) arises from Gsh2^+^ progenitors from the lateral and caudal ganglionic eminences in the ventral brain. Finally, a third wave of OPCs is generated postnatally from the dorsal Emx1^+^ progenitor and contributes to ∼80% of the OLs in the dorsal brain (Kessaris et al., 2006; Tripathi et al., 2011; Guo et al., 2021). Thus, OPCs quickly generate mature myelinating OLs within the early postnatal weeks and throughout life, but the differentiation rate declines with age. New OLs in the adult brain actively participate in myelin remodeling, and remyelination through OPC differentiation is of interest to treat demyelinating neuropathologies (Watanabe et al., 2002; Hill et al., 2013; Serwanski et al., 2018; Bonetto et al., 2020; Figure 1A). OPCs express voltage-gated ion channels and membrane receptors that give them a complex electrophysiological profile and, in contrast to other glial cells, they receive unidirectional synaptic input from neurons (Berger et al., 1991; Bergles et al., 2000; Jabs et al., 2005; Müller et al., 2009; De Biase et al., 2010; Reyes-Haro et al., 2010; Matyash and Kettenmann, 2010; Reyes-Haro et al., 2013; Arellano et al., 2016; Bedner et al., 2020; Labrada-Moncada et al., 2020). Glutamatergic inputs into OPCs processes usually derive from long-range axons, while GABAergic inputs derive from local interneurons with specifically distributed synaptic contacts. Those from fast spiking interneurons are mainly located at OPCs somata and proximal parts of the processes, and those from non-fast spiking interneurons are mainly located at the distal parts of OPCs processes (Lin and Bergles, 2004; Mangin et al., 2008; Müller et al., 2009; Mangin et al., 2012; Balia et al., 2015; Orduz et al., 2015).

## GABA_*A*_Rs are Expressed in OPCs

The γ-aminobutyric acid (GABA) is considered the main inhibitory neurotransmitter that hyperpolarizes neurons in the brain. However, GABA produces depolarization in OPCs and promotes an increase in intracellular Ca^2+^ through activation of ionotropic GABA_*A*_ receptors (GABA_*A*_Rs) (Kirchhoff and Kettenmann, 1992; Arellano et al., 2016). Indeed, *in vitro* studies reported functional expression of GABA_*A*_Rs, in both OPCs and mature OLs (Gilbert et al., 1984; Hoppe and Kettenmann, 1989; Von Blankenfeld et al., 1991), and showed that their expression in the OL membrane is controlled by their interaction with neurons (Arellano et al., 2016). The main receptor expressed *in vitro* has functional and pharmacological characteristics that distinguish it from those expressed in most neurons (Arellano et al., 2016; Ordaz et al., 2021). Investigating the molecular identity and structure-function of the GABA_*A*_Rs is important to develop pharmacological tools to act specifically on OL receptors.

GABA_*A*_R is a pentameric protein that consists of a combination of subunits coupled to a Cl^–^ channel and modulated by clinical compounds, such as barbiturates and benzodiazepines (Olsen and Sieghart, 2008). The GABA_*A*_R family includes 19 identified genes that code for the same number of subunits (α1–α6, β1–β3, γ1–γ3, δ, e, ϕ, π, and ρ1–ρ3) widely distributed in the CNS. The most common pentameric array of subunits is 2α, 2β, and 1γ, with α1β2γ2 as the main combination in neuronal synapsis (Olsen and Sieghart, 2008). GABA_*A*_Rs containing the γ2 subunits in neurons usually correspond to synaptic receptors while extrasynaptic transmission is commonly mediated through receptors containing the δ subunit (Olsen and Sieghart, 2008). The main GABA_*A*_Rs studied in the oligodendroglial lineage include combinations that contain γ subunits. However, not all of them contain the γ2 subunit, instead they contain the γ1 subunit (Ordaz et al., 2021). For example, single-cell RT-PCR studies reported that α2, α3, β3, γ1, and γ2 subunits were expressed in OPCs from the hippocampus of young mice (P17) (Passlick et al., 2013). Initially, the expression of γ subunits in OPCs was controversial because the contradictory action of benzodiazepines on the GABA response (Von Blankenfeld et al., 1991; Bronstein et al., 1998; Williamson et al., 1998). The reason for this ambiguity appears to be that the OL-GABA_*A*_R responds to benzodiazepines with classical potentiation in a neurotransmitter concentration-dependent manner (Arellano et al., 2016). Indeed, GABA concentration is critical to occlude (EC_50_ ∼ 100 μM) or promote potentiation (≤EC_30_) by benzodiazepines such as diazepam or flunitrazepam. Potentiation by benzodiazepines supports the involvement of a γ subunit in the conformation of the OL-GABA_*A*_R, at least at the neonatal stage, given that these studies were performed in primary cultures of cells isolated from the neonate forebrain (P0–P2) or OLs from the optic nerve at P10–P12 (Arellano et al., 2016). However, the OL response to GABA displayed blockage by Zn^2+^, within the μM range, and lack of modulation by indiplon (a positive modulator that acts on receptors containing the γ2 subunit), clearly indicating that the γ2 subunit was not involved in the conformation of the OL-GABA_*A*_R. The potentiation of GABA_*A*_Rs with loreclezole (an antiepileptic compound that acts as a positive allosteric modulator) suggest the involvement of β2 or β3 subunits (Wafford et al., 1994), while low sensitivity to GABA (EC_50_ ∼ 100 μM) suggests the expression of α3 subunit (Karim et al., 2013). This supports the original idea that the OL-GABA_*A*_R contains α3/β2 or β3/γ1 or γ3 subunits. This proposal was further reinforced by single cell RNAseq transcriptome studies in OPCs (NG2^+^ cells) from P17 mice (Larson et al., 2016), and by the transcriptomic analysis derived from datasets available in public domain resources, for GABA_*A*_R subunit gene expression (assessed by RNAseq) in PDGFRα^+^ cells isolated by fluorescence-activated cell sorting from mice whole brains, as well as from cortex and corpus callosum (CC) cells of adult mice (Ordaz et al., 2021). Thus, all available transcriptomic analyses support the idea that OPCs express the coding sequences for various subunits, where α2 and α3, together with β2, β3, and γ1 subunits, were well represented and highlighted the low or even null expression of γ2, γ3, β1, or δ subunits. Nevertheless, the molecular composition of GABA_*A*_R in OLs seems to change with age and probably also depends on their localization in the brain. Although there is scant information, another possible source of diversity depends on the species. In this context, a transcriptomic analysis in human OPCs (PDGFRα^+^ cells) resulted in a high expression of ε subunits (Serrano-Regal et al., 2020). The expression of γ2-containing receptors has been documented in NG2^+^ cells of the mouse barrel cortex during the first postnatal month (Vélez-Fort et al., 2010). Nonetheless, their expression is downregulated in older animals, a time-course that correlates well with a parallel decrease of neuron-OPC synaptic contact and a switch from synaptic to extrasynaptic GABAergic signaling transmission. However, studies in OPCs isolated at early stages (P0-P12) of myelination, from the forebrain and the optic nerve, indicated that a receptor devoid of the γ2 subunit is responsible for their GABA sensitivity. To explore the possible configuration of this receptor, based on its pharmacology and the available transcriptomic analyses, a heterologous expression study was carried out combining in different arrangements the subunits that have been proposed in their configuration. The study showed that the combination α3β2γ1 mimicked the characteristics of the endogenous receptor when expressed in *Xenopus laevis* oocytes. Moreover, OPC α3 subunit silencing by siRNA transfection shifted the EC_50_ for GABA (from 76 to 46 μM), while γ1 subunit silencing reduced the current amplitude by 55%, indicating their involvement in the endogenous receptor conformation (Figure 1B; Ordaz et al., 2021). A question of obvious interest that remains unresolved is whether the configuration containing the γ1 subunit corresponds to receptors located in the neuron-OPC synapse during the neonatal stage. The involvement of γ2 subunit in the conformation of synaptic GABA_*A*_Rs in neurons is a well-known fact; however, the substitution of this subunit by γ1 has been reported in some cases, and its sub-localization in the OL membrane has not been systematically explored. On the other hand, information about the pharmacological characteristics of the OL-GABA_*A*_R could provide tools that would allow a specific modulation. Thus, β-carbolines, described originally as inverse agonists acting on the benzodiazepine site, differentially and potently enhance the response in OLs when compared to those expressed by neurons (Cisneros-Mejorado et al., 2020; Ordaz et al., 2021). The potentiating effect of β-carbolines has also been demonstrated previously in different neuronal GABA_*A*_Rs (Sieghart, 2015). This effect is observed when the classic benzodiazepine site is blocked or eliminated. However, the OL-GABA_*A*_R responds directly to diverse β-carbolines applications with an enhancement of the response to GABA. Thus, the identification of β-carbolines as selective positive modulators of OL-GABA_*A*_Rs, as well as the molecular identity of the binding site, may help to study the role of GABAergic signaling during myelination (Figure 1B; Ordaz et al., 2021).

## GABA_*A*_Rs-Mediated Signaling Provides a Regulatory Pathway for OPCs Development

GABA plays an important signaling role in neurodevelopment and synaptogenesis, thus, GABAergic synaptic input to neuronal precursor cells is known to promote the survival and maturation of neuronal progenitors (Tozuka et al., 2005; Song et al., 2013), while a non-canonical function of GABA has been highlighted as a synaptogenic element shaping the early establishment of neuronal circuitry in mouse cortex (Oh et al., 2016). For example, the subventricular zone is a neurogenic niche where GFAP^+^ /nestin^+^ cells generate neuroblasts (Doetsch et al., 1999; Garcia et al., 2004). GABA is spontaneously released from neuroblasts and diffuses to activate GABA_*A*_Rs functionally expressed by GFAP^+^ /nestin^+^ cells. This signaling limits their proliferation, maintaining a balance between neuroblast production and migration in the subventricular zone (Liu et al., 2005). It has been proposed that interactions between axons and the exploratory processes of OPCs could lead to myelination in a similar way to those between dendrites and axons that eventually lead to synapse formation (Almeida and Lyons, 2014). In fact, OPCs express GABA_*A*_Rs and receive synaptic input from interneurons early in neurodevelopment (Lin and Bergles, 2004; Zonouzi et al., 2015; Labrada-Moncada et al., 2020). The activation of GABA_*A*_Rs induces membrane depolarization and [Ca^2+^]_*i*_ elevation in OPCs and pre-myelinating OLs (Tong et al., 2009; Arellano et al., 2016; Labrada-Moncada et al., 2020), similar to what has been observed in immature neurons (Ben-Ari et al., 2007). However, the mechanism induced by activation of GABA_*A*_Rs in OPCs involves Na^+^ influx through non-inactivating Na^+^ channels, which in turn triggers Ca^2+^ influx via Na^+^/Ca^2+^ exchangers (NCXs). This unique Ca^2+^ signaling pathway is further shown to be involved in the migration of OPCs (Tong et al., 2009). The [Ca^2+^]_*i*_ increase promotes differentiation and survival of OPCs through voltage-gated calcium channel CaV1.2 activation (Pitman et al., 2020), and controls their migration through influx via NCXs (Tong et al., 2009). Thus, OPC depolarization by GABA has multifactorial consequences. For example, incubation of the GABA_*A*_R agonist muscimol (100 μM) in primary cultures of OPCs decreased the number of BrdU^+^/OPCs, suggesting that GABA signaling can directly influence their proliferation (Zonouzi et al., 2015). Moreover, loss of GABA_*A*_R-mediated synaptic input to OPCs by hypoxia seems to promote the proliferation of these cells and a delay in OL maturation resulting in cerebellar white matter (WM) demyelination during the early postnatal stage (Zonouzi et al., 2015). Recently, we explored whether GABAergic signaling included other glial cells within the cerebellar WM (Labrada-Moncada et al., 2020). The cellular composition of WM is dominated by glial cells and axons, and neuronal somata represents less than 1% of the cells (Sturrock, 1976; Reyes-Haro et al., 2013). First, using calcium imaging analysis we tested the effect of the GABA_*A*_R agonist muscimol (50 μM) on cerebellar WM cells and found that 39% of them responded with an intracellular Ca^2+^ increase. No response to baclofen was observed, suggesting that GABA-mediated Ca^2+^ signaling occurs through GABA_*A*_Rs at early postnatal development (P7–P9). Then, astrocytes were labeled with sulforhodamine B (SRB) and we observed that muscimol responding cells did not incorporated SRB. To further explore the identity of these cells, electrophysiological analysis was made. In agreement with Ca^2+^ imaging studies, muscimol did not generate any current response in the recorded astrocytes indicating lack of the functional expression of GABA_*A*_Rs. In contrast, muscimol-mediated currents were elicited in NG2^+^ cells, indicating that OPCs were the main cell type in cerebellar WM electrically responsive to GABA through the activation of GABA_*A*_Rs (Labrada-Moncada et al., 2020).

Altogether, these observations strongly suggest that GABA_*A*_R-mediated signaling represents a specific regulatory pathway to control migration, proliferation and maturation of OPCs during early postnatal development of the cerebellar WM.

## Therapeutic Potential of GABA_*A*_Rs in Remyelination

Premature infants (23–32 weeks gestation) are at high risk of developing diffuse white matter injury (DWMI), a leading cause of neurodevelopmental disabilities often linked to chronic hypoxia (Back, 2006; Anjari et al., 2009). DWMI is also known as bilateral periventricular leukomalacia and is associated with subcortical WM damage characterized by a marked loss of OPCs (Back et al., 2001, 2002) resulting in important behavioral, cognitive and motor deficits (Allin et al., 2008; Larroque et al., 2008). GABAergic signaling markers are reduced in the cortex and WM of preterm infants diagnosed with DWMI (Robinson et al., 2006), and studies have also reported a reduction of cortical GABA concentration in a pre-clinical mouse model of DWMI (Komitova et al., 2013). A therapeutical strategy to overcome demyelination is to enhance OPC proliferation and maturation to improve functional outcomes (Scafidi et al., 2014).

In this context, GABAergic signaling was tested on the myelination rate in a DWMI murine model induced by neonatal hypoxia treatment. First, cerebellar WM hypomyelination was revealed by electron microscopy and immunolabeling with myelin basic protein (MBP) and neurofilament (NF200) antibodies. This was accompanied by an increase in OPC proliferation (Ki67^+^ /Olig2^+^ cells) and a decrease in mature OLs (CC1^+^ cells). OPCs also showed a reduced GABAergic synaptic input from interneurons. Second, GABA_*A*_R-mediated signaling was tested with a pharmacological approach *in vivo* where the administration of bicuculline, a selective GABA_*A*_R antagonist, increased the number of OPCs by threefold but decreased the amount of mature OLs. In contrast, tiagabine and vigabatrin, inhibitors of the GABA transporter and the GABA transaminase, respectively, decreased OPCs proliferation and increased the number of mature OLs. Thus, enhancing GABA availability by administration of tiagabine and vigabatrin ameliorated the effects of hypoxia and resulted in oligodendrogenesis enhancement and progression of OPCs to myelinating OLs (Zonouzi et al., 2015).

## Neurosteroids and GABA_*A*_Rs in Remyelination

The peripheral benzodiazepine receptor (PBR) is expressed in peripheral organs and regulates the transport of cholesterol to mitochondria for the synthesis of pregnenolone, a progesterone-derived neurosteroid that is metabolized to allopregnanolone (ALLO) (reviewed by Gavish et al., 1999), a positive allosteric modulator of GABA_*A*_Rs that is produced *de novo* by both neurons and glial cells, and acts with 20-fold higher potency than benzodiazepines and barbiturates (Majewska et al., 1986; Purdy et al., 1991; Belelli and Lambert, 2005; Sripad et al., 2013). During late pregnancy, high levels of ALLO maintain the increased GABAergic tone required to attenuate the hypothalamic pituitary-adrenal axis activation and suppress the stress response (Neumann et al., 1998; Brunton et al., 2014). A recent study, performed in pregnant and postpartum rats, used lysolecithin-induced demyelination in the CC to explore a correlation between GABAergic signaling and remyelination (Kalakh and Mouihate, 2019). The results showed augmented proliferation of OPCs and myelination index in the demyelinated CC of pregnant rats when compared to virgin and postpartum rats. Furthermore, Western blot studies showed higher expression of myelin oligodendrocyte glycoprotein (MOG) and 2′,3′-Cyclic nucleotide 3′-Phosphodiesterase (CNPase) in focally demyelinated CC from pregnant rats. Thus, it seems that pregnancy generates a pro-myelinating environment in response to a focal demyelination injury (Kalakh and Mouihate, 2019). To test if the increased GABAergic tone associated with pregnancy was involved, bicuculline was injected together with lysolecithin. Bicuculline administration worsened the demyelination lesion and reduced OPC density in pregnant rats, suggesting that GABA_*A*_R-mediated signaling promotes remyelination. Since increased GABAergic tone is modulated by ALLO during pregnancy, the contribution of endogenous ALLO on de/re-myelination was tested by inhibiting its synthesis with finasteride, an inhibitor of 5α-reductase. The administration of finasteride also resulted in a large demyelination lesion and the reduction of OPC population compared to vehicle-treated pregnant rats, in a similar manner to the results obtained with GABA_*A*_R antagonism (Kalakh and Mouihate, 2019). Immunofluorescence analysis showed that the expression of GABA_*A*_R-γ2 subunit was absent in the saline-injected CC of pregnant, virgin or postpartum rats. However, a subset of OPCs (NG2^+^ cells) in the vicinity of the demyelination lesion were immunoreactive to this subunit, and Western blot studies showed an increased expression in the CC of pregnant rats when compared to virgin or postpartum animals (Kalakh and Mouihate, 2019). Overall, these results suggest that GABA_*A*_Rs containing the γ2 subunit are upregulated in OPCs during remyelination in adult animals. Altogether, these results suggest that OPC proliferation may be promoted through ALLO-modulated GABA_*A*_Rs in the demyelinated CC of pregnant rats.

In another study testing the promyelinating action of ganaxolone, a synthetic analog of ALLO with increased bioavailability, was used in an experimental model of preterm birth (Shaw et al., 2019). Brain WM volume is reduced in preterm children, which correlates with an increased risk of developing attention deficient hyperactivity disorder (AHDH) and anxiety. Fetal neurodevelopment requires exposure to neurosteroids provided by the placenta during pregnancy, and preterm birth is accompanied by a drastic drop of ALLO. This was experimentally reproduced in guinea pigs after *in utero* administration of finasteride. The administration of ganaxolone to preterm guinea pigs improved myelination of the CA1 region of the hippocampus and subcortical WM, suggesting that GABA_*A*_R modulation by neurosteroids may be a potential therapeutical tool to overcome myelination deficits in early neurodevelopment (Shaw et al., 2019).

## β-Carbolines and GABA_*A*_Rs in Remyelination

The β-carbolines are part of a heterogeneous family of compounds found in several fruits, tobacco, alcohol and coffee, among others. They are also present in the mammalian cerebrospinal fluid and brain (for review see Polanski et al., 2011). β-carbolines have been assessed in behavioral tests due to their potential modulatory effect on GABA_*A*_R and success in a variety of illnesses (Cowen et al., 1981; Novas et al., 1988; Medina et al., 1989; Rowlett et al., 2001; Venault and Chapouthier, 2007). In addition to the inverse agonist action with a negative effect, it has been reported that β-carbolines act on a second binding site with a positive modulatory effect (Novas et al., 1988; Sieghart, 2015).

In some GABA_*A*_Rs this effect seems to involve a low-affinity binding site described for diazepam (Walters et al., 2000; Sieghart, 2015). Indeed, N-butyl-β-carboline-3-carboxylate (β-CCB) acts on OL-GABA_*A*_Rs with a strong enhancement on the GABA response (Arellano et al., 2016), which is not observed in neuronal cells isolated from brain cortex (Cisneros-Mejorado et al., 2020). Thus, β-CCB action as a positive modulator of GABAergic neuron-OL signaling was tested on remyelination using a murine model of demyelination/remyelination (Woodruff and Franklin, 1999; Cisneros-Mejorado et al., 2020). Ethidium bromide was stereotaxically injected into the caudal cerebellar peduncle (c.c.p.) of rats to induce demyelination, and the resulting lesion was histologically characterized with black-gold II staining (BGII) and longitudinally characterized by magnetic resonance imaging (MRI) to detect microstructural changes (Cisneros-Mejorado et al., 2020). As expected, decreased fractional anisotropy (FA) and increased radial diffusivity were evident following c.c.p. lesioning. The MRI analysis correlated well with a decrease in myelin content as revealed by BGII staining. However, when systemic β-CCB was administered daily for 2 weeks in lesioned animals, an increase in the FA was observed in parallel with a radial diffusivity decrease. These changes also correlated with recovery of myelin staining with BGII (Figure 1C). Animal behavior was unaffected by β-CCB as revealed by open field exploration, freezing, signs of pain, anxiety or apparent aggression. These observations strongly suggest remyelination enhancement by β-CCB treatment.

## Discussion

GABA_*A*_R-mediated signaling plays a key role during embryonic and early postnatal neurodevelopment of OPCs. Indeed, GABA_*A*_R activation is involved in the regulation of proliferation, differentiation, axon-glia recognition and myelination onset (Zonouzi et al., 2015; Arellano et al., 2016; Hamilton et al., 2017). A subset of OPCs prevail in the adult brain; these cells are known as NG2 glia and functional expression of GABA_*A*_R-mediated signaling has also been reported (Vélez-Fort et al., 2010). Experimental evidence from different studies suggests that GABA_*A*_R-mediated signaling to OPCs is important to improve myelination or remyelination in demyelinating diseases (Zonouzi et al., 2015; Kalakh and Mouihate, 2019; Shaw et al., 2019; Cisneros-Mejorado et al., 2020). Specific control and/or targeting of GABA_*A*_Rs expressed in the OPCs will help to understand their role in its physiology, and particularly to comprehend the role of GABAergic signaling in the myelination/demyelination/remyelination process of the brain. In this direction, β-carbolines and neurosteroids, particularly β-CCB and ALLO, are promising therapeutical candidates to selectively target OL GABA_*A*_Rs and promote remyelination.

## Author Contributions

DR-H, AC-M, and RA contributed to the manuscript, approved the submitted version, and designed the content of the manuscript. All authors contributed to the article and approved the submitted version.

## Conflict of Interest

The authors declare that the research was conducted in the absence of any commercial or financial relationships that could be construed as a potential conflict of interest.
